# Pig Slurry Management Producing N Mineral Concentrates: A Full-Scale Case Study

**DOI:** 10.1021/acssuschemeng.2c07016

**Published:** 2023-03-22

**Authors:** Axel Herrera, Giuliana D’Imporzano, Elisa Clagnan, Ambrogio Pigoli, Elena Bonadei, Erik Meers, Fabrizio Adani

**Affiliations:** † Gruppo Ricicla - DiSAA, Università degli Studi di Milano, Via Celoria 2, 20133 Milan, Italy; ‡ O.B. Di Orazio Brunelli e Figli − S.N.C., Via Adua 52, 25034 Orzinuovi, BS, Italy; § Department of Green Chemistry and Technology, Faculty of Bioscience Engineering, University of Ghent, Coupure Links 653, 9000 Ghent, Belgium

**Keywords:** Life cycle assessment, Nutrient recovery, Reverse osmosis, Water recovery, Pig slurry

## Abstract

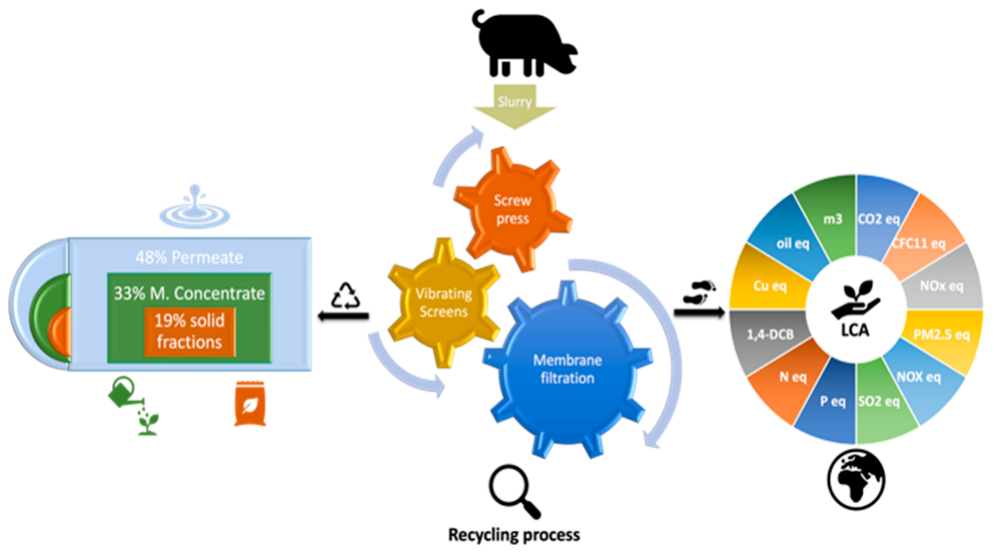

Manure treatment to recover nutrients presents a great challenge to delocalize nutrients from overloaded areas to those needing such nutrients. To do this, approaches for the treatment of manure have been proposed, and currently, they are mostly under investigation before being upgraded to full scale. There are very few fully operating plants recovering nutrients and, therefore, very few data on which to base environmental and economic studies. In this work, a treatment plant carrying out full-scale membrane technology to treat manure to reduce its total volume and produce a nutrient-rich fraction, i.e., the concentrate, was studied. The concentrate fraction allowed the recovery of 46% of total N and 43% of total P. The high mineral N content, i.e., N-NH_4_/total-N > 91%, allowed matching the REcovered Nitrogen from manURE (RENURE) criteria proposed by the European Commission to allow the potential substitution of synthetic chemical fertilizers in vulnerable areas characterized by nutrient overloading. Life cycle assessment (LCA) performed by using full-scale data indicated that nutrient recovery by the process studied, when compared with the production of synthetic mineral fertilizers, had a lower impact for the 12 categories studied. LCA also suggested precautions which might reduce environmental impacts even more, i.e., covering the slurry to reduce NH_3_, N_2_O, and CH_4_ emissions and reducing energy consumption by promoting renewable production. The system studied presented a total cost of 4.3 € tons^–1^ of slurry treated, which is relatively low compared to other similar technologies.

## Abstract 2

Manure processing reduced waste storage volume by recovering water and producing recycled derived fertilizers/amendments.

## Introduction

Industrial livestock production carries increasing challenges due to the excessive nutrient loads of the slurry produced and the potential environmental problems which it creates.^1,2^ Based on lifecycle analyses, animal farming can be responsible for up to 18% of global greenhouse gas (GHG) emissions.^3^ The use of manure as fertilizer can heighten environmental pollution through the release of ammonia (NH_3_), nitrous oxide (N_2_O), and nitric oxide (NO) into the atmosphere and through the leaching of nitrate (NO_3_^–^), nitrite (NO_2_^–^), and ammonium (NH_4_^+^) to groundwater and surface water bodies.^4,5^ Additionally, when fertilization is managed on nitrogen (N) crop requirements, it generally results in significant phosphorus (P) overload, as manure tends to have low N:P ratios, causing further water eutrophication.^6^ Poor N and P use efficiencies in agriculture in the past, and the consequent water contamination, forced EU authorities to create regulations to limit the use of animal slurries; i.e., they set the application rate limit of slurry at 170 kg N ha^–1^ in nitrate vulnerable zones (NVZ) (Nitrate Directive Guidelines, Council Directive 91/676/EEC).^7^ This directive became an integral part of the EU Water Framework Directive, one of the key directives protecting waters from agricultural pressures (Council Directive 2000/60/EC).

However, other agricultural regions with low livestock densities and scarcity of nutrients may require a greater use of mineral fertilizers to increase production yields. Nonrenewable natural resources (e.g., phosphate rock, oil, and natural gas) are needed to produce chemical fertilizers. Considerable negative environmental impacts and high costs are related to the extraction of raw materials, manufacture, and use of these fertilizers.^8−10^ Therefore, better geographical redistribution of animal slurry nutrients to be used as fertilizer could efficiently and economically reduce chemical fertilizer consumption, especially in those areas characterized by low livestock densities.^11^ Moreover, the current climate change crisis calls for better management practices, suggesting nutrient recycling from biowaste by paying attention to both the environment and the costs of recovery, transformation, and usage of biowaste.^12^

The Circular Economy has gained attention in the past decade by encouraging new upcycling nutrient practices, followed by the adopting of new Fertilizing Products Regulations (EC/2019/1009).^13,14^ For instance, the recent REcovered Nitrogen from manURE (RENURE)^15^ criterion was proposed by the EU Joint Research Centre as a suggestion for recovering animal slurry nutrients to overcome the barriers that hindered the safe use of recovered products, allowing the use of N over the limits in NVZ indicated by the Nitrate Directive. RENURE criteria require that recovered products from manure should have a mineral nitrogen content higher than 90% of the total N, opening the door to a potential substitution of synthetic chemical fertilizers.^15^

Slurry management is commonly guided by an initial physical separation into liquid and solid fractions that facilitate the transport of nutrients at reduced weight and volume.^16,17^ Though the liquid fraction is still enriched in nutrients, it does not guarantee a high nutrient recovery efficiency, and its volume is still large due to the high water content. Therefore, further post-treatment technologies are necessary to separate/recover clean water and concentrate nutrients into separate products for better management while reducing their volume.^18^ Membrane technologies based on reverse osmosis (RO) are extensively used for water and wastewater treatment by producing pure water for reuse and a nutrient-rich liquid (concentrate); however, their application on animal effluents has been limited.^19^ One of the main limitations is fouling and membrane clogging due to the accumulation of unwanted materials present in the infeed that can reduce flow speed and membrane performance, leading to high energy consumption and cost.^20^ High energy consumption can lead to high GHG emissions from nutrient recycling processes,^21^ making these processes unsustainable from an environmental point of view.

Life cycle assessment (LCA) has become an essential tool for better characterization and decision support in assessing the environmental performance of emerging technologies.^22^ A noted LCA study^23^ in the production of manure-derived fertilizer (mineral concentrate) by using RO technology has shown that emissions (e.g., NH_3_, CH_4_) coming from manure processing, derived fertilizer storage, and the use of the derived fertilizers were crucial parameters affecting the impact results of such processes. At the same time, outcomes from the environmental performance can be similar or even lower in some impact indicators compared to conventional manure management. Other reports on RO applications highlight the need for both full-scale testing and further optimization to achieve the standards for legal discharge of the cleaned water obtained into shallow waters^24^ and the importance of improvement in pretreatment technology and operation to minimize fouling while producing high-quality concentrates.^25^ Therefore, system optimization and validation of the recovered end products are essential in future studies to address improvement to meet new quality standards.^19^

The present study monitored a full-scale pig slurry treatment system (OB-Slurless) operating in Northern Italy (Lombardy). This plant was among demo cases chosen within the H2020 EU project, i.e., NUTRI2CYCLE - *Transition toward a more carbon and nutrient efficient agriculture in Europe*, No. 773682, to demonstrate the feasibility of nutrient recycling from manure, closing the C, N, and P loops. This system is based on a series of mechanical separations and concentration steps. It uses RO technology to recover water while concentrating nutrients in separated solid and liquid fractions that will ease their management and allocation.

In particular, the study analyzed the entire process of producing different fractions, evaluating processes performance and the chemical composition of the different recovered products with particular attention to N, P, and potassium (K) recovery. In addition, a life cycle assessment (LCA) was performed to assess the environmental impact of such a process, in order to compare the process sustainability with that of synthetic mineral fertilizers.

## Materials and Methods

### System Analyzed

**Figure 1:**
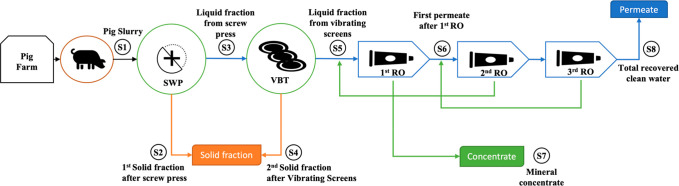
Process scheme and different fractions produced during pig slurry treatment (S1–S8): screw press (SWP), vibrating screens (VBT), and reverse osmosis (RO) sections.

The system under study (OB-Slurless) is represented by a full-scale facility located in the province of Bergamo in the north of Italy. The technology (TRL 9) follows a “plug and play” approach by being a preassembled containerized plant (i.e., in shipping containers of 12.2 m × 2.44 m × 2.59 m), requiring no more than 200 m^2^ of surface for its installation. It can treat any kind of livestock manure continuously and automatically under an extensive range of conditions. In the case under study, it treats a total raw input of 120 m^3^ day^–1^ by processing 37,800 tons of pig slurry per year. The facility started to operate in the autumn of 2020. The raw slurry (S1) (Figure 1) comes from an adjacent pig farm with a complete production cycle of 38,000 pigs from weaners and fatteners. The process follows a series of separation and concentration steps (Figure 1) by starting with a mechanical separation using a screw press (SWP) (slurry separator Cri-Man SM300, Correggio, Reggio Emilia) producing a solid fraction (S2) and a liquid fraction (S3), followed by a vibrating screening (VBT) (screening opening 0.114 mm mesh, Vibrotech, S. Antonino Di Casalgrande, Reggio Emilia) operating on fraction S3 to better refine the solid removal from the liquid fraction (S3), producing a new liquid fraction (S5) and a solid fraction (S4) that is joined to fraction S2.

Then, the liquid fraction (S5) coming from the last separation enters the first stage of reverse osmosis (1st RO), performed by vibratory shear enhanced processing (VSEP). This system uses torsional vibration of the membrane surface, which creates high shearing forces, reducing fouling and polarization of the membrane.^26^ This step allows retaining most of the nutrient content in a concentrate (S7) solution, ready to be stored and exported due to the reduced volume. The clarified fraction (S6), or first permeate, follows the subsequent RO steps (2nd and 3rd RO) using extra-fouling resistant 8 in. spiral membranes allowing, in combination with 1st RO, to get a permeate (recovered freshwater) (S8) that can be reused for cleaning or safe discharge in the environment.

Furthermore, the system integrates a system platform (called RILoB) for continuous remote monitoring of performance to better control processing fluxes and clean-in-place (CIP) membranes. For instance, the CIP runs automatically for all RO stages at fixed times based on the need for washing set by operational experience (i.e., 1st RO, once per day; 2nd RO and 3rd RO, every 10–15 days). The number of washes performed and the quantities of chemical products used in the washes are recorded both on the system panel and on the platform. The integration of informatic technologies for better process management to prevent fouling and provide maintenance when needed are some of the practices which can extend the life span of membranes (up to 3 years). Besides giving a suitable pretreatment to the RO input (e.g., optimal mechanical separation), these practices are vital to achieving high-quality end products and extending the system’s operational life.

### Chemical Characterization of Raw Slurry and Processing Stage Products

Raw slurry and the different fractions obtained (i.e., S1–S8) were sampled three times over six months of the observation period (January – Winter, May – Spring, and July – Summer of 2021). During each sampling event, homogenized samples (2000 mL each divided in four samples) were collected from the different separation stages at noon. The samples were collected in polyethylene sampling containers and transported within 2 h from the facility to the laboratory in cooler boxes filled with ice. Samples were stored at 4 °C. Each sample was tested in triplicate.

The following parameters were measured: dry matter at 105 °C (DM 105 °C), dry matter at 600 °C (DM 600 °C),^27^ and total Kjeldhal nitrogen (TKN) (EN 13652);^28^ ammonia-N (NH_4_-N) (ISO 5664 method);^29^ nutrients (P, K, Ca, Mg, Fe, Mo, Mn), heavy metals (Cd, Cr tot, Ni, Pb, Cu, Zn, Hg, Al), and microelements (As, Co, Se) according to DIN EN ISO 11885^30^ and UNI-EN 16174.^31^ Elemental analyses were carried out by using an inductively coupled plasma mass spectrometry (ICP-MS, Varian Inc., Fort Collins, CO, USA).

### Pathogen DNA Screening

Samples for molecular analyses were collected on three occasions: January 14 (Winter), May 12 (Spring), and July 8 (Summer) of 2021. Liquids and solids were collected in sterile bottles from six sample sites, i.e., raw slurry (S1), solid fractions after screw press (S2), and after vibrating screen (S4), first permeate (S6), concentrate (S7), and final permeate (S8). After collection, samples were transported cooled to the lab (within 2 h), where they were processed within the same day.

DNA extraction was performed for each sample. In order to collect total DNA (both intra and extracellular), liquid samples were first subjected to ethanol precipitation.^32,33^ Briefly, 10 volumes of a fresh sample (15 mL for S1 and S7 and 500 mL for S6 and S8) were mixed with 1 volume of 3 M sodium acetate and 23 volumes of absolute ethanol and stored at −20 °C overnight. Samples were centrifuged (10,000 rpm for 15 min at 4 °C), supernatants discarded, and pellets washed with 70% ethanol and air-dried. On the second and third campaigns, 15 L were collected for locations S6 and S8 and filtered through 0.2 nm filters (Ahlstrom-Munksjö, Germany) in order to check only for intracellular DNA. From each sample, DNA was extracted in 3 replicas using the DNeasy PowerSoil Kit (Qiagen, Germany) according to manufacturer’s instructions. Yield and purity (A260/A280 and A260/A230) of the extracted DNA were quantified on a Nanodrop 1000 spectrophotometer (Thermo Fisher Scientific), while eventual fragmentation was determined through gel electrophoresis 1% (w/v) 1 × TAE agarose gels. Extracted DNA was stored at −80 °C until analyses.

Following manufacturer’s protocol for low-abundance microbial species, samples were screened through the 96-well (48 × 2) format Microbial DNA qPCR Array for water analysis (Qiagen, USA) on an Applied Biosystems 7300 Real-Time PCR System for the presence or absence of 45 bacterial pathogens or indicators of fecal contamination targeting 16S rRNA and virulence genes (eae, stx2A, stxA). This assay includes, among the others, pathogens highlighted as a cause of significant risks for health and of important relative infectivity (i.e., *Campylobacter* spp.; enterohemorrhagic *Escherichia coli* virulence factors: *eae*, *stx2A*, and *stxA*; *Helicobacter pylori*; *Legionella pneumophila*; *Mycobacterium avium* and *intracellulare*; *Shigella dysenteriae* and *Vibrio cholera)* (OJEC, 2000; WHO, 2011; EPA, 2021).^34−36^ The assay further contains two positive “Pan Bacteria” controls that target bacteria universally and an additional positive PCR control to detect possible inhibition and efficiency (see manufacturer’s manuals for a complete list of target characteristics). Triplicates were then merged, and DNA concentration of the samples was standardized as required. The assay requires a minimum of 250 ng of DNA per sample. Only the S6 and S8 spring samples resulted below this threshold; 2500 ng was used for all samples except for S6 and S8 in winter and summer, where 250 and 450 ng were used, respectively, due to DNA extraction yields. PCR cycling conditions were an initial incubation at 95 °C for 10 min followed by 40 cycles of denaturation at 95 °C for 15 s and annealing and extension at 60 °C for 2 min with FAM fluorescence used for detection. Laboratory procedures were carried out in a sterile PCR hood with PCR grade reagents and plasticware. Negative, positive, and inconclusive results were calculated following the manufacturer’s protocol. Results were reported semiquantitatively using an inverse cycle threshold. Statistical differences among sampling sites and across time were assessed through a nonparametric Kruskal–Wallis test followed by Dunn’s Test for multiple comparisons on R studio (version 4.1.2).

### Evaluating Separation Efficiency

The separation efficiency of the solid–liquid fractions was estimated in the major processing steps (i.e., screw press, vibrating screen, and RO) by using a simple separation index (*E*_*t*_) based on Svarovsky, 2000^37^ (eq 1). The index is defined as the simple ratio of the total mass of the solids or nutrients separated to the total mass of solids or nutrients fed into the separator, where *U* and *Q* are the masses (kg) of the concentrated/solid and slurry feed streams, respectively. While *M*_*c*_ and *S*_*c*_ are the concentrations (g kg^–1^ fresh weight) of a component (*c*) in the respective streams: solid (*M*) and feed slurry (*S*). The simple separation index ranges between 0 and 1, where, for example, *E*_*t*_ = 0.50 indicates the presence of 50% of *c* in the solid fraction. *E*_*t*_ is presented as a percentage in the further data results.
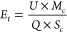
1
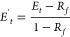
2

When a diluted concentrated/solid stream is noticeable, a “reduced” efficiency concept is used to look at the net separation effect by considering the total mass split by the separator into the solid and feed streams. Therefore, the reduced separation index (*E′*_t_) (eq 2) is expressed by the simple total efficiency defined in eq 1 and *R*_*f*_ = *U*/*Q*, as the solid fraction to total slurry ratio. Equation 2 satisfies the requirements for a net efficiency designation because it gives zero (0) for conditions of no separation when *E*_*t*_ = *R*_*f*_ and one (1) for complete separation of solids when *E*_*t*_ = 1. The reduced separation index ranges from −1 to 1, where positive values indicate an increase in the concentration of *c* in the solid fraction compared with the raw slurry, and negative values indicate an increase in the concentration of *c* in the clarified liquid fraction.^38^

### Environmental Impact Assessment

An LCA was conducted to assess impacts in producing recovered nutrients from the concentrate (S7) (CON scenario). The functional unit studied was the production of 50.4 N tons y^–1^ (as NH_4_-N), 20.1 P tons y^–1^, and 31.5 K tons y^–1^, i.e., the nutrient contents of the concentrate produced in a year: 12,600 tons. This scenario was compared with a reference scenario (REF) which included the production of chemical synthetic fertilizers, i.e., urea: 109.5 tons y^–1^, triple superphosphate (20% P content): 100.3 tons y^–1^, and potassium chloride: 61.2 tons y^–1^. N efficiency was considered equal for both scenarios studied because of the concentrate’s characteristics (i.e., mineral N/total N ratio ≥ 90%) that made it similar to Haber–Bosch-derived chemical N fertilizer.^15,39^

The LCA was attributional, with a cradle-to-gate approach; i.e., the system boundaries included all the slurry processing steps, the storage of the products, and the transport of mineral concentrate to the fields. As the facility is located within the pig farm, it does not require transport for the slurry collection. The data inventory was based mainly on primary data obtained from the facility (Table 1). A large part of the resources used by the system are chemicals for membrane cleaning and pH adjustment, besides the primary energy and water consumption. The facility’s construction was based partly on the study by Al-Sarkal and Arafat (2013)^40^ for a treatment RO plant. Other capital goods related to the mechanical separation, containerized to the system in ship containers of 12.2 m, and storage of the final product (S7) by a circular concrete tank were included by using literature and databases (Ecoinvent V3.5),^41^ all considering a lifespan average in the structure of 20 years. Emissions from processing and transfer storage were considered for NH_3_ and CH_4_ based on Schils et al. (2015)^42^ and Brockmann et al. (2014).^43^ A default emission factor of 0.01 kg N_2_O-N per kg of ammonia nitrogen emitted for an indirect source of N_2_O emissions was considered as proposed by IPCC (2006).^44^ Emissions from the concentrate storage were not included as they were supposed to be negligible because of the sealed concrete floors and assumption of covered storage.^23,45^ The release to the environment (water bodies) of permeate and the corresponding emissions (i.e., NH_4_, P, and heavy metals) were also considered. The reference scenario (REF) was composed of the production of urea, triple phosphate, and potassium chloride, with transport included to a regional storehouse. Therefore, a minimum transfer distance of 100 km from the storehouse to the area where synthetic mineral fertilizers are used was assumed for REF; the Ecoinvent v3.5 2018 data were used to quantify transportation impacts.

**Table 1:** Main Data Inventory of the System Studied

INPUTS				
Parameter	Unit	Quantity	Data source	Additional info.
Pig slurry	tons y^–1^	37,800	Provided by the facility	Main waste handled by technology
Water (Aqueduct)	m^3^ y^–1^	24	Provided by the facility	Only for manual and automatic cleaning operations during maintenance
Electricity	kWh y^–1^	285,120	Provided by the facility	Energy needed to run the technology (36 kWh)
Acid product (Citric acid 2%)	L y^–1^	4380	Provided by the facility	Chemical needed for membrane cleaning
Alkaline product (Sodium hydroxide)	L y^–1^	4380	Provided by the facility	Chemical needed for membrane cleaning
Sulfuric acid H_2_SO_4_	L y^–1^	1000	Provided by the facility	For pH adjusting
Sodium hydroxide	L y^–1^	1000	Provided by the facility	For pH adjusting
Mineral oil	L y^–1^	6	Provided by the facility	For lubrication

OUTPUTS				
Parameter	Unit	Quantity	Data source	Additional info.
Permeate: Clean water	m^3^ y^–1^	18,000	Provided by the facility	Permeate density 1.00 t m^–3^
Liquid fraction: Mineral concentrate	tons y^–1^	12,600	Provided by the facility	Concentrate density 1.05 t m^–3^
Solid fraction: (S2 + S4)^a^	tons y^–1^	7200	Provided by the facility	Solid dry matter around 22%
Transport	km	40	Provided by the facility	Distance covered by hauliers to supply concentrate and synthetic fertilizers
Membranes disposed at end of life	kg y^–1^	460	Provided by the facility	Calculated on a 3-year service life of all type of installed membranes
Mineral oil	L y^–1^	6	Provided by the facility	For lubrication
**Emissions from processing**				
Indirect dinitrogen monoxide (N_2_O-N)	kg tons^–1^	0.0012	IPCC 2006^46^	Treatment and outside storage
Ammonia (NH_4_-N)	kg tons^–1^	0.12	Schils et al., 2015;^42^ De Vries et al., 2012^47^	Treatment and outside storage
Methane (CH_4_-C)	kg tons^–1^	0.037	Brockmann et al., 2014;^43^ Loyon et al., 2007^48^	Treatment and outside storage

a Represented by the grouping of fractions; S2 and S4 presented in Figure 1.

Transport distances, which are especially relevant for energy consumption and CO_2_ emissions, of 40 km were taken for both scenarios using transport, lorry, Euro 5, for the provision of nutrients to the crop fields for the CON scenario. Lastly, waste treatment disposal for the RO membranes was considered, as they are made of organic polyamide in a composite thin film. The inventory of the primary data used in the assessment is presented in Table 1.

Data were processed by using the software SimaPro Analyst 9.0.0.41.^49^ The evaluation method used was the ReCiPe 2016^50^ midpoint method under a Hierarchist perspective (H) (version 1.13), covering 18 midpoint impact indicators.

## Results

### Mass Balance and Separation Efficiency

This section provides an overview of the system with particular reference to mass balances and nutrient distribution in the different fractions (Table 2), setting the input, i.e. pig slurry, to 100% fresh weight (fw).

**Table 2:** Relative Mass Distribution for End Fractions in Terms of Percentage for Total Mass (M), Total solids (TS), Total Nitrogen (TKN), Ammonium (NH_4_^+^-N), Organic Nitrogen (ORG-N), Total Phosphorus (P), and Total Potassium (K), Assuming a Starting Content of Each Parameter Equal to 100^a^

Parameter	Raw slurry (S1)	Solid fraction (S2) disposable^b^	Solid fraction (S4) disposable^b^	Concentrate (S7) exportable^c^	Permeate (S8) disposable^d^	Total	Deviation^e^
M	100	15	4	33	48	100	–
TS	100	69	9	19	0	97	–3
TKN	100	27	5	46	0	78	–22
NH_4_-N	100	13	4	65	0	82	–18
ORG-N	100	51	6	17	0	74	–26
P	100	46	6	43	0	95	–5
K	100	18	4	69	0	91	–9

a Read the mass balance horizontally, i.e., the sum of each fraction should be 100, unless for deviation.

b Direct land application.

c Export or direct land application.

d Discharged to waterbodies.

e Negative sign indicates missing quantities in the balance.

The mass balance (all data are reported as % fw of starting pig slurry) showed that solid fractions, i.e., S2 and S4, after SWP and VBT separations represented 15% and 4% of the original slurry, respectively. The use of the SWP led to a high performance and to the concentration, in fraction S2, of most of the TS (69%), organic N (51%), and P (46%) that are initially present in pig slurry. This device allowed a solid/liquid separation cheaper than the one obtained by centrifugation because the use of flocculants is avoided preserving membrane integrity and reducing energy consumption. Performance was close to the top range separation index (*E*_*t*_ = 15%) found for this type of separator, in terms of volume (Table 3), according to other studies (11%–14%)^18,38^ and also connected to the highly reduced retention for TS (*E′*_*t*_ = 0.64), P (*E′*_*t*_ = 0.37), and organic N (*E′*_*t*_ = 0.43). Instead, the solid fraction (S4) coming from the VBT separator contained only 9% of the TS, 9% of organic N, and 6% of P. These values reflected the lower VBT separation performance, i.e., *E′*_*t*_ = 0.17 for TS than SWP, i.e. , *E′*_*t*_ = 0.64, and the fact that it has a polishing function as a secondary separator. The vibrating screen makes a more fine solid/liquid (S/L) separation, holding particles between 0.1 and 0.03 mm, operating in a low range of separation efficiency depending on the screen opening.^51^

**Table 3:** Separation Performance in Terms of Simple Separation Index (*E*_*t*_) and Reduced index (*E′*_*t*_) for Main Separation Steps in the Process

	*E*_*t*_ (%)	Reduced separation efficiency index (*E′*_*t*_)					
Separator/membrane	Mass	TS	TKN	NH_4_-N	Organic N	P	K
Screw press	15	0.64	0.14	–0.02	0.43	0.37	0.03
Vibrating screen	4.7	0.17	0.01	0.00	0.04	0.04	0.01
1st reverse osmosis membrane	41.2	0.73	0.67	0.77	0.55	0.3	0.86

RO infeed (S5) was represented by a liquid fraction from the second separator (VBT). It represented 81% of the total mass, holding 23% of slurry TS, and a still significant part of the major nutrients characterizing the pig slurry, i.e., 57%, 73%, and 74% of the slurry N, P, and K, respectively. The first RO separator determined a reduction of 59% of the S5 mass, within a 4% decrease in the TS content (19%), and concentrating N (46%), P (43%), and K (69%) all in one-third (S7) of the slurry mass. These concentrations agreed with the high simple separation index found for the first membrane separator (*E*_*t*_ = 41%) and were linked to the reduced separation efficiencies (*E′*_*t*_) for N (0.67), P (0.3), and K (0.86). The subsequent two RO steps (2nd RO and 3rd RO) produced permeate as a final product (S8) that contained almost half (47.6%) of the slurry mass and counted less than 1% in the balance for all the elements. In general, the mass distribution of recovered products was in line with other studies using RO filtration,^23^ with purified water counting for 42%–50% of the total mass, 33%–39% of the total mass as a nutrient concentrate, and the remaining (19% of total mass) as a solid fraction.

Regarding the fate of the primary nutrients, N ended almost half in the concentrate (46%), mainly as ammonia, resulting in a high NH_4_^+^-N/TKN ratio (91%), while 32% of TKN was retained in the solid fractions, mainly as an organic form. The 22% of TKN was missing in the global balance, probably because of sampling uncertainties and NH_3_ losses during the different processing steps. Thus, RO separation performance for N (*E′*_*t*_ = 0.67) did not show such a high separation index as other studies reported.^52^

Total P distribution comprised 52% in the solid fractions, while the remainder was allocated in the concentrate. Although P is associated with smaller particles and low solubility in the liquid fraction, the lack of chemical pretreatment and the fact that at least 50% of P is bonded to fine particles (<25 μm)^38^ allow the first RO filtration to achieve high retention (43%). However, RO separation efficiency did not outperform for P, compared to other studies (*E′*_*t*_ higher than 0.5),^26,52^ possibly due to the recirculation from the second RO membrane, resulting in a high P concentration in S5. Regarding K, its high retention (76%) in the concentrate was caused by its high solubility in water; so RO was very effective in separation for K as other studies have found^23,52^ and was effectively reflected in the high separation efficiency index (*E′*_*t*_)= 0.86 achieved.

### Chemical Composition of Slurry and End Products

Chemical characterization of the different separated solid and liquid fractions is shown in Table 4. Pig slurry characteristics (S1) were in the range reported for a complete cycle of fattening/farrowing pigs.^53^ Total solid detected was of 54.2 ± 6.3 g kg^–1^; N, that was mainly found as inorganic N, was 1085 ± 908 mg kg^–1^ fw. Again, K was 1232 ± 363 mg kg^–1^ fw, and P was 1295 ± 534 mg kg^–1^.

**Table 4:** Chemical Properties of Different Solid (SF) and Liquid (LF) Fractions in the Processing Steps^a^

		Pig Slurry	SF from SWP^b^	LF from SWP	SF from VBT^c^	LF from VBT	LF after 1st RO Stage	Concentrate	Permeate
Parameter	Unit	S1^d^	S2	S3	S4	S5	S6	S7	S8
Total solids (105 °C)	g kg^–1^	54.2 ± 6.3	252.5 ± 31.8	26.5 ± 4.7	117.6 ± 1.8	15.2 ± 2	0.1 ± 0	31 ± 4	0.01 ± 0
Volatile solids (600 °C)	g kg^–1^	42.6 ± 1.9	229.1 ± 31.8	21.1 ± 7.2	104.4 ± 1.2	10.8 ± 0.3	0.1 ± 0	11.8 ± 2.7	**0**
Total Kjeldhal N	mg kg^–1^	3015 ± 832	5393 ± 1154	2620 ± 60	3345 ± 187	2103 ± 653	538 ± 613	4120 ± 531	4 ± 1
NH_4_-N	mg kg^–1^	1930 ± 257	1675 ± 425	1838 ± 547	1937 ± 273	1786 ± 230	224 ± 127	3750 ± 583	4 ± 1
Organic N	mg kg^–1^	1085 ± 908	3718 ± 1043	782 ± 527	1408 ± 407	317 ± 860	180 ± 695	565 ± 377	**n.d.**^e^
NH_4_-N/Total N	%	68 ± 20	32 ± 7	70 ± 20	58 ± 11	96 ± 42	115 ± 94	91 ± 6	100 ± 0
P	mg kg^–1^	1295 ± 534	3988 ± 811	1042 ± 59	1829 ± 498	1169 ± 276	200 ± 21	1660 ± 53	1.53 ± 0.24
K	mg kg^–1^	1232 ± 363	1440 ± 85	1070 ± 241	1280 ± 116	1135 ± 14	143 ± 10	2530 ± 305	0.93 ± 0.23
Na	mg kg^–1^	733 ± 470	687 ± 45.6	463 ± 200	703 ± 144	677 ± 195	170 ± 4	880 ± 115	**0**
Mg	mg kg^–1^	534 ± 187	942 ± 50	353 ± 69	466 ± 131	301 ± 14	22 ± 2	488 ± 48	**0**
Al	mg kg^–1^	63 ± 53	72 ± 5	27 ± 12	42 ± 17	23 ± 8	5 ± 0	29 ± 2	**0**
Ca	mg kg^–1^	1806 ± 1196	4052 ± 1139	1042 ± 379	1660 ± 693	846 ± 126	91 ± 7	1321 ± 54	1.5 ± 0.3
Cr	mg kg^–1^	1.1 ± 0.6	1.4 ± 0.2	0.376 ± 0.001	0.77 ± 0.15	0.52 ± 0.21	0.14 ± 0.02	**n.d.**	0.000517 ± 0.00001
Mn	mg kg^–1^	20 ± 13	41 ± 13	11 ± 4	**n.d.**	8 ± 1	n.d.	**n.d.**	0.0152 ± 0.0033
Fe	mg kg^–1^	77 ± 24	190 ± 49	52 ± 20	78 ± 46	37 ± 10	3.43 ± 0.7	60 ± 7	**0**
Ni	mg kg^–1^	1.26 ± 0.57	2.6 ± 1.7	n.d.	**n.d.**	1.63 ± 0.33	n.d.	1.38 ± 0.86	**n.d.**
Cu	mg kg^–1^	12 ± 11	25.3 ± 5.9	8.8 ± 1.9	**n.d.**	7.53 ± 0.27	n.d.	8.3 ± 0.5	0.0097 ± 0.0036
Zn	mg kg^–1^	20.7 ± 2.1	40.3 ± 7.4	21.62 ± 0.06	32.3 ± 4.9	21.94 ± 0.91	2.11 ± 0.33	24.1 ± 0.7	0.02763 ± 0.00083
Se	mg kg^–1^	0.226 ± 0.083	**n.d.**	0.099 ± 0.01	**n.d.**	n.d.	n.d.	**n.d.**	**n.d.**
Mo	mg kg^–1^	0.519 ± 0.081	**n.d.**	0.43 ± 0.21	**n.d.**	0.46 ± 0.13	n.d.	**n.d.**	**n.d.**
Cd	mg kg^–1^	n.d.	**n.d.**	n.d.	**n.d.**	n.d.	n.d.	**n.d.**	**n.d.**
Ba	mg kg^–1^	1.84 ± 0.43	4.77 ± 0.36	1.19 ± 0.19	2.5 ± 1.3	1.39 ± 0.6	0.43 ± 0.13	2.15 ± 0.11	0.0016 ± 0.00014
Pb	mg kg^–1^	0.31 ± 0.11	0.34 ± 0.13	0.15 ± 0.02	**n.d.**	0.28 ± 0.13	0.09 ± 0.001	0.25 ± 0.11	**n.d.**

a Data are presented based on wet weight; end products are emphasized in bold.

b SWP: Screw press section.

c VBT: Vibrating screen section.

d Different separation sections (S1–S8) are presented in Figure 1.

e n.d.: not detected.

The first solid fraction (S2) coming from the SWP separator showed high TS and VS contents, i.e., 252 ± 31 and 229 ± 31 g kg^–1^, respectively. The high solid content brought high organic N, P, and Ca concentrations (referred to fresh matter), i.e., 3718 ± 1043, 3988 ± 811, and 4052 ± 1139 mg kg^–1^, respectively. These characteristics agreed with other reports using SWP.^54^ The second solid fraction (S4) coming from the VBT separator, showed different characteristics with respect to the S2 fraction, with TS less concentrated than formerly, i.e., 117.6 ± 1.8 and 104.4 ± 1.2 g kg^–1^, respectively. Organic N, P, and Ca contents were of 1408 ± 407, 1829 ± 498, and 1660 ± 693 mg kg^–1^, respectively.

**Figure 2:**
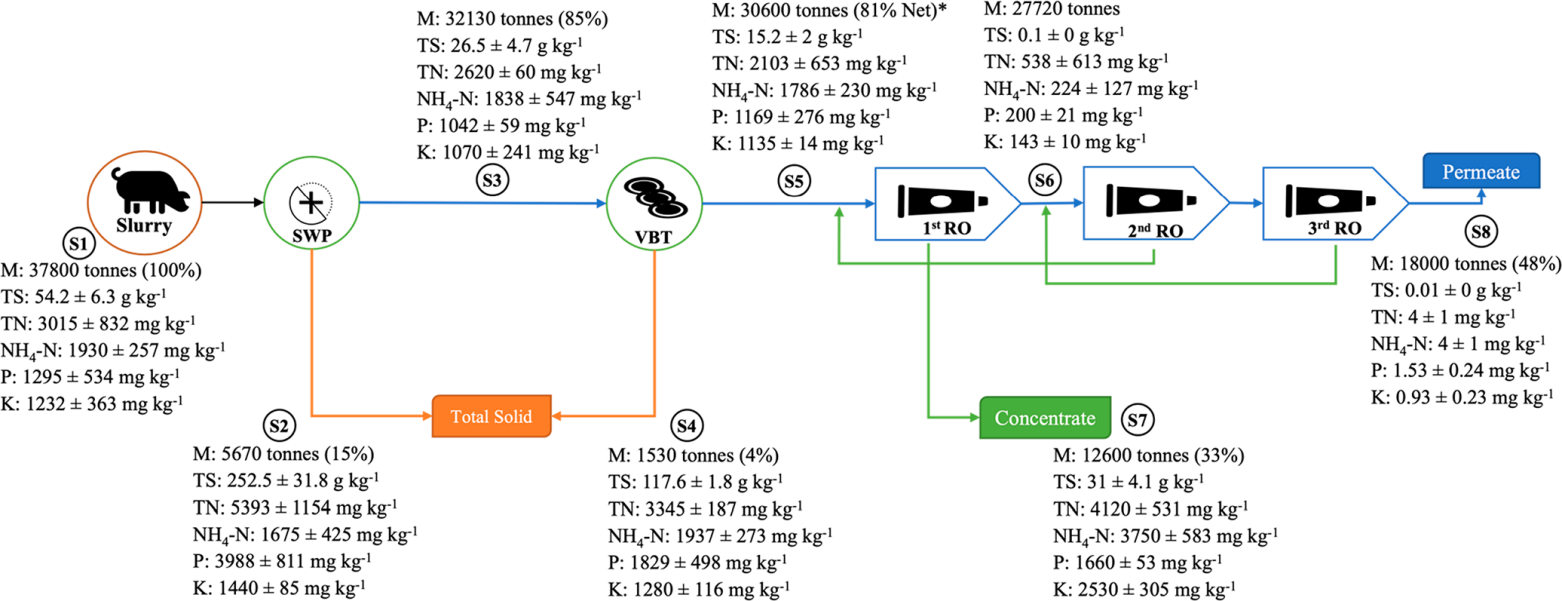
Mass balance and flow: mass (M), total solids (TS), total nitrogen (TN), total ammonium (NH_4_-N), phosphorus (P), and potassium (K); all values refer to fresh weight. *Net value calculated without taking into account recirculation coming from the 2nd RO stage. Fractions legend (S1–S8): S1= pig slurry; S2 = 1st solid fraction after screw press; S3 = liquid fraction from screw press; S4 = 2nd solid fraction after vibrating screens; S5 = liquid fraction from vibrating screens; S6 = first permeate after 1st RO; S7 = mineral concentrate; S8 = total recovered water.

The second largest product, the concentrate (S7), contained most of the ammonium (3750 ± 583 mg kg^–1^), that represented 91% of TKN (4120 ± 531 mg kg^–1^) and a large part of P and K, i.e., 1660 ± 53 and 2530 ± 305 mg kg^–1^, respectively. Other elements, such as Na (880 ± 115 mg kg^–1^) and Mg (488 ± 48 mg kg^–1^) were almost comparable to the concentrations in the infeed slurry but less variable. In comparison with other RO concentrates produced from pig manure previously described,^25,42,52^ S7 presented relatively low contents of TKN and K, with a high presence of P (related to the N:P ratio). These differences among concentrates compositions can differ from system to system (e.g., use of coagulants/flocculants, pretreatment steps, type of membrane), as well as the characteristics of the infeed slurry. For the main final product, as the permeate (S8), N was present only in the mineral form at a low concentration, i.e., 4 ±1 mg kg^–1^, while P and K were found to be 1.5 ± 0.2 and of 0.9 ± 0.2 mg kg^–1^, respectively. Figure 2 encapsulates the mass flow distribution along the different fractions described above.

Heavy metals such as Pb, Ba, Ni, Cr, and Mn were found at high concentrations mainly in the solid phase (S2), as previously reported.^55^ However, the same metals through the filtration process were reduced in concentrations to less than 2.2 mg kg^–1^ or not detected (Mn and Cr) in fraction S7. The permeate (S8) showed the clearest reduction of the same metals, to less than 0.015 mg kg^–1^ or they were not detected (Ni and Pb), while other metals such as Zn and Cu showed a similar trend of reduction as Cr and Mn with final concentrations of 0.02 and 0.009 mg kg^–1^, respectively. Organically bound and carbonate precipitated metals are the largest fractions of metals within slurry^56,57^ explaining the fact why metals concentrate more in fractions with high organic matter content, i.e., solid fractions, and therefore to a lesser extent in the concentrate.

### Presence of Pathogens

The DNAs from enteric and pathogenic bacterial species and virulence markers were detected by qPCR in 12 out of 18 samples (Figure S1). As expected, results obtained showed that pig slurry (S1) was characterized by a high level of bacterial DNA, including pathogenic forms that are commonly found in fecal material.

The most common bacterial DNAs found were related to *Arcobacter butzleri*, *Desulfovibrio desulfuricans*, *Lactobacillus gasseri*, *Ruminococcus obeum*, and *Citrobacter freundii*, which are all indicators of fecal contamination being commonly found in mammal gastroenteric tracts. In particular, *A. butzleri*, *D. desulfuricans*, and especially *C. freundii* are also human pathogens correlated with infections and mainly gastrointestinal diseases. Other bacteria retrieved (such as *Aeromonas spp.*, *Campylobacter spp.*, etc.) are identified as highly important and emerging pathogens in drinking water.^34−36^

The permeates (S6 and S8), which represented the end points for the liquid fractions with S8 directly discharged into shallow water, did not reveal the presence of pathogenic DNA.

For the scope of this study, a semiquantitative qPCR approach was used in order to characterize the potential infectivity of a wide array of pathogens; i.e., only the presence of DNA was detected. In order to quantify the real risks of infectivity, pathogen cultivation must be performed in order to assess the presence of viable pathogens, their abundance, and the compliance with water standard limits. Since these monitoring programs are cost intensive, they are limited to a low number of indicators that are further reduced by the small number of effectively culturable bacteria. On the other hand, the simple detection of the presence of pathogenic DNA highlighted the necessity for the adoption of preventive and protective measures to reduce infection risks.

### Environmental Assessment

**Figure 3:**
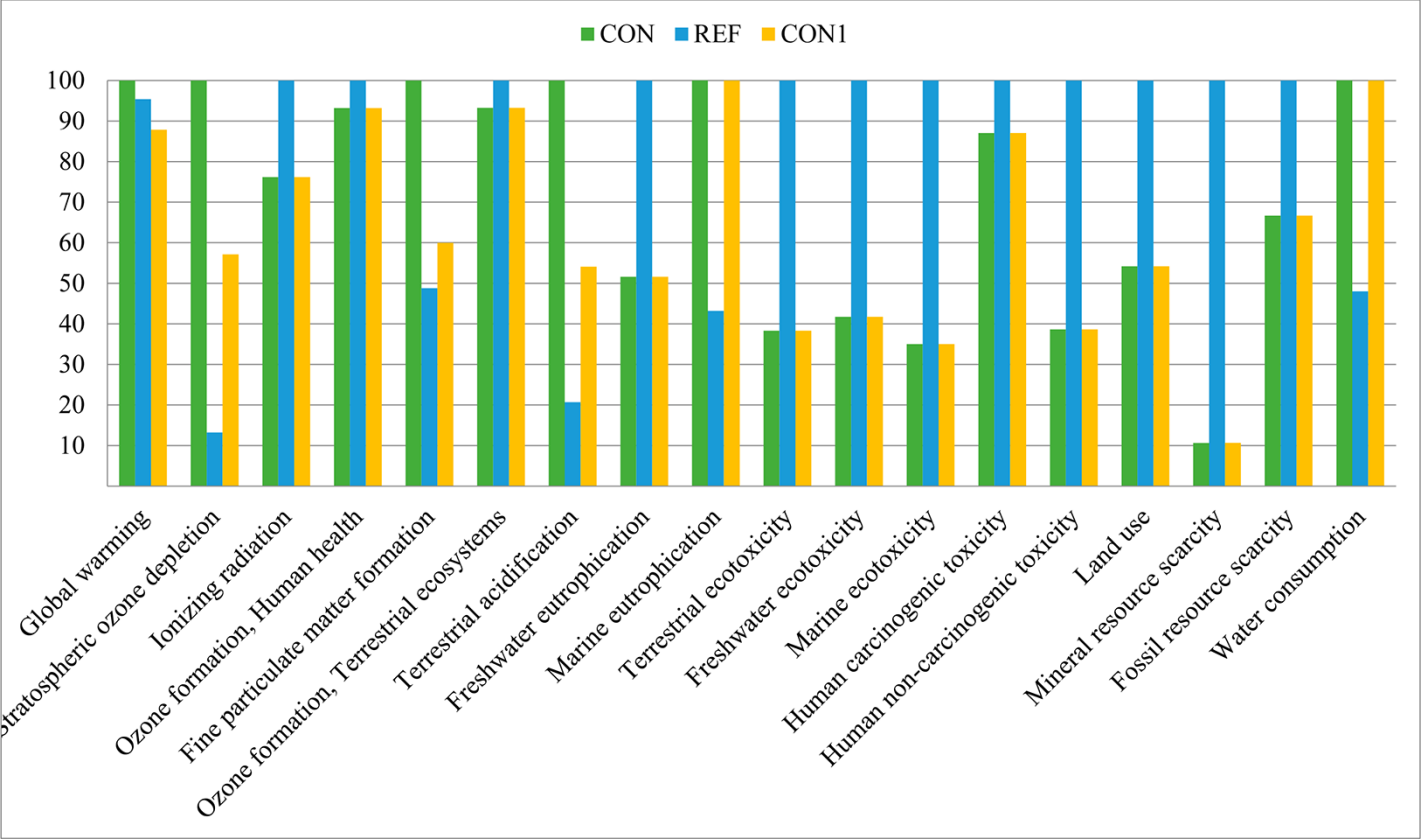
Comparative environmental results for Scenario CON, Scenario REF, and the alternative proposed Scenario CON1 (using plastic film to cover storage) (ReCiPe 2016 Midpoint (H) V1.03/World (2010) H/Characterization method). Global warming potential (GWP).

Results for the impact in the 17 midpoint categories are presented in Table 5; the characterization values are also presented on a relative percentage attributing a value of 100 to the highest value reported for each category (Figure 3). The system studied (CON) compared to the reference scenario (REF), showed lower impacts for 12 categories, with better performance (<50%) for the ecotoxicity group, followed by the resources depletion category, except for water consumption. Other categories related to ozone formation and ionizing radiation had a comparable or closer impact on the REF scenario (>80%). In contrast, the REF scenario did better in particulate matter formation, ozone depletion, terrestrial acidification, and global warming; details of the most relevant categories are explained as follows.

**Table 5:** Impact Category Values for Two Evaluated Scenarios CON and REF^a^

Impact category	Unit	Scenario CON	Scenario REF
Global warming	kg CO_2_ equiv	2.9 × 10^05^	2.8 × 10^05^
Stratospheric ozone depletion	kg CFC11 equiv	0.77	0.102
Ionizing radiation	kBq Co-60 equiv	3.0 × 10^04^	3.9 × 10^04^
Ozone formation, Human health	kg NOx equiv	549	589
Fine particulate matter formation	kg PM2.5 equiv	1.4 × 10^03^	685
Ozone formation, Terrestrial ecosystems	kg NO_X_ equiv	561	602
Terrestrial acidification	kg SO_2_ equiv	1 × 10^04^	2.0 × 10^03^
Freshwater eutrophication	kg P equiv	77.1	149
Marine eutrophication	kg N equiv	25.4	11
Terrestrial ecotoxicity	kg 1,4-DCB	3.8 × 10^05^	1 × 10^06^
Freshwater ecotoxicity	kg 1,4-DCB	2.8 × 10^03^	6.7 × 10^03^
Marine ecotoxicity	kg 1,4-DCB	4.2 × 10^03^	1.2 × 10^04^
Human carcinogenic toxicity	kg 1,4-DCB	7.7 × 10^03^	8.8 × 10^03^
Human noncarcinogenic toxicity	kg 1,4-DCB	9.1 × 10^04^	2.3 × 10^05^
Land use	m^2^ a crop equiv	2.1 × 10^03^	4 × 10^03^
Mineral resource scarcity	kg Cu equiv	576	5.4 × 10^03^
Fossil resource scarcity	kg oil equiv	6.7 × 10^04^	1 × 10^05^
Water consumption	m^3^	1.1 × 10^06^	5.4 × 10^05^

a Impact assessment calculated according to ReCiPe 2016 Midpoint (H) V.1.1. Functional Unit: N, P, and K produced from mineral concentrate in one year.

Climate warming potential (GWP) represents the increase of radiative forcing because of greenhouse gases (GHGs, expressed as a kg CO_2_-eq), showing an impact for the CON scenario of 2.9 × 10^05^, which was higher by 5% than the REF scenario. CON impact was mainly due to electricity consumption, slurry emissions from processing and storage, and transportation, i.e., 57%, 24%, and 15% of the total, respectively. Instead, for the REF scenario, urea (natural gas use) and triple superphosphate production determined almost its total impact, i.e., 60% and 33%.

### Eutrophication

From marine eutrophication, expressed as a kg N eq , the CON scenario showed a more significant impact, more than double compared to that calculated for REF, since the release of recovered water containing ammonium in the environment explained about 83% of the effect. Although ammonium content levels were below legal limits, <15 mg l^–1^, its impact was relevant due to the large amount of water released into the environment. In the case of freshwater eutrophication (kg P eq ), CON was lower by 58% than REF. The REF scenario showed a higher impact as the production of triple superphosphate determined in large part the impact (75%) because of the P release in waterbodies, while in the CON scenario the impact was only due to electricity and related P emissions due to combustion.

### Toxicity

Toxicity expressed as a kg 1,4-DCB-eq (1,4-dichlorobenzene-equivalents) represents the fate and impact of chemical emissions. The ecotoxicity group (marine, freshwater, and terrestrial) indicators had a lower impact for the CON scenario (35%–42%), more than half lower compared with REF. This impact was explained mainly by electricity and transport (60%–70% of total impact) and did not outweigh the production of chemical fertilizers, i.e., 33%–59% for urea and 28%–53% for P fertilizer (range). Human toxicity, the noncarcinogenic class, had a similar response as previous ecotoxicity indicators, with an impact that was 40% lower for CON than REF, explained by the same process as above. In the case of the carcinogenic class, the CON scenario had an impact 13% lower than REF, where transport (26%), electricity (42%), and appliances used (i.e., shipping containers and tank storage) (30%) explained the lower impact of CON.

### Resource Depletion

For the CON scenario, mineral resources presented a considerably lower impact (11%), followed by land use (54%) and fossil resources (67%), values that were not comparable to the resources demanded by the production of chemical fertilizers (REF). In contrast, water depletion (m^3^) represented a higher impact (52%) for CON than REF; even when there was a large amount of water recovered in the environment, it represented a savings of only −1.6%, compared to electricity consumption which explained 97% (1.1E06 m^3^) of the high water use in CON, a process that needs more energy than that consumed by the production of fertilizers in REF.

### Terrestrial Acidification and Ozone Formation

Terrestrial acidification (expressed as kg SO_2_-eq ) was five times higher for CON than REF because of ammonia emissions during slurry processing. Photochemical ozone formation was similar for the two scenarios: about 93% of the impact was explained mainly by NO_X_ emissions during electricity production and transport.

### Other Categories

Ionizing radiation expressed as a kBq Co-60 eq was lower in CON (24% lower compared to REF), and it was explained mainly by electricity consumption (86%), transport (11%), and chemicals used for membrane cleaning and pH adjusting (1%). Conversely, the REF scenario performed better for fine particulate formation (kg PM2.5 equiv) and ozone depletion (kg CFC11 equiv) with a lower impact than CON, i.e., 51% and 87% less, respectively. The higher effect found in CON was explained by NH_3_ and N_2_O emissions during processing, contributing to 80% and 96% separately for each impact category.

## Discussion

### Processed Product and Its Environmental Performance

The final product obtained from the process described and discussed above, i.e., the mineral concentrate, was characterized by a high NH_4_-N/NTK ratio, i.e., 91%, which allows it to be classified as RENURE. Nevertheless, low total N content suggests for the future an appropriate post-treatment (e.g., ammonia stripping) to increase N concentration in the final products. Previous works have shown that by integrating the ammonia stripping step with reverse osmosis,^52,58^ the total N concentration can be increased by almost 10 times (N = 61 g kg^–1^ fw). Concentrate showed very good fertilizing properties, not only because of N content but because potassium was present in a concentration that was about double than that of the initial slurry. P was also well represented as it was in a concentration comparable to that of the pig slurry, because the system did not use any coagulant/flocculants (e.g., salts of calcium or aluminum that precipitate P) to improve the separation efficiency of the raw slurry. In this case, the concentrate produced is more suitable for those areas characterized for P application limitation or for P-poor soils.

The production of mineral concentrates from manure processing leads to better performances in terms of environmental quality (i.e., reduced raw materials needs and toxicity) due to the upcycling of nutrients directly from pig manure. On the other hand, the energy required for its processing can exceed the reduction in energy achieved from the minor impact due to transportation, increasing GWP, ozone depletion, and particulate matter formation. Studies by Lopez-Ridaura et al., 2009,^59^ and De Vries et al., 2012,^23^ pointed out that the categories of climate change, terrestrial acidification, and particulate matter are more heavily impacted (+10%–30%) by the processing of manure into mineral concentrate rather than its direct use in agriculture when credits from avoided chemical fertilizer use are being accounted for. This higher impact reported was explained by the release of GHGs due to the energy needed for manure processing and storage, as was found in this study.

When considering an alternative scenario (CON1) that uses an impermeable plastic film covering manure storage able to reduce NH_3_, N_2_O, and CH_4_ by 50% (Kupper et al., 2020^60^) the impacts decreased significantly by at least 40% concerning CON, for the terrestrial acidification, particulate matter formation, and ozone depletion categories (Figure 3). Furthermore, GWP from CON1 was 13% lower than CON; the low benefit was obtained because this category was primarily affected by electricity consumption and transport. The employment of these improved management solutions within the CON1 scenario achieved impacts similar to the REF scenario, which is relevant in the process optimization context. Following the suggestion proposed in the CON1 scenario, the facility actually started implementing the covering of transfer tanks at the end of the second year of operation.

Another factor to be considered in improving the environmental impact of the proposed manure processing is the energy consumption (representing 55% in GWP), which was attributed mainly to the constant pumping in the membrane filtration stage among first RO to third RO steps because of the need of high shear and cross-flow velocities to minimize membrane fouling. When considering the energy demand of the system, i.e., 7.5 kWh m^–3^ of slurry treated (i.e., production of 1.33 kg of N from mineral concentrate), it was relatively lower than standard membrane filtration systems; i.e., 10–20 kWh m^–3^,^61^ and of energy consumption reported for urea, i.e., 8.36 kWh kg^–1^.^62^ Despite the low energy demanded by the system, energy consumption represented a critical factor in expanding the membrane application because of its implication to define the economic performance.^24^ Some studies suggested energy improvements by operating at low fluxes, by using enzymatic pretreatment,^63^ and integrating the use of renewable energy sources.^64^ In this case, previous LCA studies showed that the use of the solid fraction as feedstock to feed anaerobic digestion (AD),^65^ producing renewable energy by a CHP unit, improved the whole system; i.e., the use of solid fraction as feedstock reduced GHGs emissions and fossil fuel needs by 10% and 21%, respectively.

From a microbial perspective, as expected, the initial slurry was characterized by the presence of DNA belonging to bacterial species commonly used to monitor fecal contamination and water quality.^34,35,66^ Although the use of a PCR-based method indicated the presence of potential risk, the RO process was an effective treatment for pathogens removal as permeate phases (i.e., S6 and S8) showed the absence of any markers, indicating that VSEP membranes and the spiral-wound element with polyamide thin-film composite membranes was effective as a sterilization method. On the other hand, other fractions still contain pathogens (DNA), and they could be potentially infectious. Treating pig slurry by AD could provide a partial sanitation of slurry further reducing risks.

Additionally, the second and third RO stages ensure control over the reduction of N and heavy metals. This follows Vaneeckhaute et al., 2011,^24^ recommendations on including two more filtration stages after VSEP filtration to meet the criteria for safe permeate discharge in shallow water. Therefore, the permeate met quality standards by the absence of pathogenic markers and heavy metals, besides having parameters for controlling surface water quality, such as N and P levels under the legal thresholds (i.e., ≤15 and ≤10 mg L^–1^). All these characteristics comply with the legal limits stated by the European legislation (91/271/CEE), concerning urban wastewater treatment for its safe discharge into the surface.

### Economic Value of Concentrate as a Recovered Fertilizer

The OB-Slurless system presented a total cost of 4.3 € tons^–1^ of treated slurry, a relatively low value compared to other similar technologies using RO post-treatment (Table 6). By looking at the details of this cost, membrane replacement occupied the largest expense, i.e., 1.8 € m^–3^ slurry treated, followed by electric energy, i.e., 1.5 € tons^–1^ slurry treated, chemical products, i.e., 0.62 € tons^–1^ slurry treated, and labor for ordinary maintenance, i.e., 0.27 € tons^–1^. In general, the low demand for input resources and labor from the OB-Slurless unit is associated with the automated monitoring process to prevent and maintain the lives of membranes, besides the low energy demand (7.5 kWh tons^–1^). For instance, when considering the added value that can result from the postproduction of ammonium sulfate, as mentioned above, the overall total specific cost for a stripping system can vary from 2–8.1 € tons^–1^,^67^ where the investment cost for a stripping unit (100 m^3^ day^–1^) is estimated at 750,000 €, with an amortization corresponding to 1.58 € tons^–1^. The specific cost for energy power is 1.06 € tons^–1^, and chemicals used in the stripping process are soda or Ca(OH)_2_ for pH adjusting and sulfuric acid, for which costs are estimated at 1.5 € tons^–1^.^68^ Thus, the total cost will be around 5 € tons^–1^. Knowing that, a good quality ammonium sulfate (6%–7% N, 30% ammonium sulfate) can have an expected market value of 50–120 € tons^–1^,^52,67^ a price which is more than 10 times higher than that for N/K concentrates (1.2 € tons^–1^).^67^ This represents an important revenue, especially when this scrubbing salt is part of the top priority materials in the RENURE frame, with many studies supporting its equivalent performance to that of synthetic N fertilizers.^69,70^

**Table 6:** Cost and Energy Demand by Different Systems Using RO Post-Treatment, Total N, P, and K Recovered in Retentate (or Concentrate), and Percentage of Water Recovered Per Unit of Feedstock Treated

System (recovered fertilizer product)	Feedstock treated	Total cost (€ ton^–1^)	Energy demand (kWh ton^–1^)	Total N recovered (kg ton^–1^)	P recovered (kg ton^–1^)	K recovered (kg ton^–1^)	Water recovered (%)	Reference
OB-Slurless (Mineral Concentrate)	Pig slurry	4.3	7.5	1.32	0.52	0.82	48	This study
GENIUS (Concentrate RO1)	Digestate mainly from livestock manure	21	22	2.5	0.044	2.5	18	Van Puffelen et al., 2022^71^
Double cartridge RO (RO–Centrate)	Digestate from pig slurry and energy crops	6.9	18.5	0.93	0.048	–	46	Bolzonella et al., 2018^68^
	Digestate from cow manure and energy crops	6.9	18.5	0.57	0.07	–	43	Bolzonella et al., 2018^68^
Ama Mundu Tech. Pilot 1 (Retentate from 1st RO step)	Digestate from chicken manure, food waste, and agriculture residues	–	11.6	1.54	0.046	0.82	11	Adam et al., 2018^72^
N-free (RO Concentrate)	Digestate from swine manure	4.2	–	1.5	0.031	1.5	49	Ledda et al., 2013^52^
	Digestate from cattle manure	4.2	–	0.85	0.004	1.42	36	Ledda et al., 2013^52^
Co-Digestion plant + M. filtration (RO Concentrate)	Digestate from pig manure and corn silage	–	23	1.18	0.017	1.7	48	Chiumenti et al., 2013^73^
Wageningen Livestock Research (RO Concentrate)	Pig and dairy cattle manure	–	7.8–11.5	3.8	0.085	4.7	42	De Vries et al., 2012; De Vries et al., 2011^23,74^

As prices for synthetic fertilizers are increasing and nutrient resources are depleting, reusing valuable nutrients has enormous potential. So recycled fertilizers accompanied by the new EU Fertilizing Products Regulation (FPR) will guarantee the agronomic quality of EU-(biobased) fertilizer products while safeguarding environmental safety and human health.

This will set up a standard to promote better quality and facilitate their marketing, contributing to the circular economy.

### Mineral Concentrate vs Untreated Pig Slurry: Effect on Nitrogen Farm Management

The major value associated with this plant is the treatment of slurry to obtain an N-concentrate material that meets the RENURE criteria. The compliance with RENURE standards will allow more flexibility in the use of N derived from animal slurry. The EU Commission is currently evaluating the opportunity to allow the use of RENURE as mineral N fertilizer according to crop needs beyond the N limits of 170 kg ha^–1^ set by the Nitrates Directive.

To understand the effect of producing and using RENURE, two scenarios were compared, one using untreated pig slurry (Scenario 1) while the other using treated pig slurry (Scenario 2). Both these scenarios consider 37,800 tons of pig slurry to be managed in a farm of 289 ha of surface located in a nitrate vulnerable zone (NVZ) cropped with corn requiring 280 kg ha^–1^ N. A N efficiency of 1 is considered for chemical fertilizer and mineral concentrate, while for pig slurry (Scenario 1) and solid fraction (Scenario 2), the N efficiency is equal to the amount of ammonia content (as % of total N). Processing energy was calculated considering the electrical consumption (7 kWh) to treat 1 ton of pig slurry and considering the average electric efficiency generation in EU (0.47).^75^ Results obtained are summarized in Table 7.

**Table 7:** Comparison of Scenario 1 (No treatment) and Scenario 2 (Treatment)

Parameter	Unit	Scenario 1 (No treatment)	Scenario 2 (Treatment)
Total N from slurry to be managed	kg	113,967	113,967
N from slurry applied (according to NVZ limit)	kg	49,059	0
N from slurry solid fraction applied (according to NVZ limit)		0	35,636
N from chemical fertilizer applied	kg	49,399	0
N from mineral concentrate applied	kg	0	72,954
			
Total N distributed for fertilization	kg	98,458	108,590
N surplus from animal (to be exported)	kg	64,908	0
Readily available nitrogen distributed (ammonia or urea)	kg	80,803	80,765
Total volume distributed (SF and MC)	ton	0	19,656
Total water discharged	ton	0	18,144
Total manure managed on farm land	ton	16,272	37,800
			
Total amount of slurry exported (70 km)	ton	21,528	
Total energy needed to produce chemical fertilizer	MJ	1,234,966	0
Total energy needed for transport (slurry export)	MJ	1,137,776	0
Total energy needed for field distribution	MJ	831,600	432,432
Total energy needed for processing	MJ		2,026,723
			
Total primary energy	MJ	3,204,342	2,459,155

In Scenario 1, due to the NVZ limit (170 kg ha of animal nitrogen), 21,528 ton of slurry must be exported (average distance of 70 km), and 49,339 kg of chemical fertilizers must be supplied to the fields to meet the crop needs. In Scenario 2, the solid fraction resulting from slurry treatment can be used for sowing within Nitrate Directive limits (170 kg ha^–1^), while the mineral concentrate (RENURE) could be used instead of mineral fertilizers in top dressing (exceeding the NVZ limits), therefore avoiding the need of chemical fertilizers.

The total primary energy is lower for Scenario 2 (Table 7) than for Scenario 1, even when considering an electricity generation of low efficiency for average EU standards.

In conclusion, the possibility to use mineral concentrate (RENURE), exceeding the Nitrate Directive limits, could make the farm self-sufficient in terms of N-fertilizer supply and could eliminate the need to export material outside the farm area and to buy mineral N fertilizers.

## Conclusions

Implementing the membrane separation system in pig slurry management significantly reduced the waste storage volume by recovering water and producing two recycled-derived fertilizers/amendments. The concentrate which is the second largest process product (33%), presents RENURE characteristics, allowing the export of N and P from the farm (46%, and 43%, respectively, of the initial input), reducing nutrient pressure. The energy demand to process slurry (7.5 kWh ton^–1^) determines most of the impacts, and the further optimization of energy efficiency can significantly improve the environmental performance of the process. Indicators related to the depletion of resources and toxicity show a much lower impact due to slurry upcycling than the raw resources demanded by synthetic fertilizers production. The system has a low-resource demand, and thus, it is economically competitive in the market with similar technologies.

## Supporting Information Available

Enteric and pathogenic bacterial species and virulence markers screening in the main solid–liquid fractions (Figure S1) (PDF)
