# Supplementary material for: Pig Slurry Management Producing N Mineral Concentrates: A Full-Scale Case Study
Source: ACS Sustain Chem Eng. 2023 Mar 22;11(19):7309–22. doi: 10.1021/acssuschemeng.2c07016 (PMC10196920; doi:10.1021/acssuschemeng.2c07016)
Supplement: Supplementary file 1 — sc2c07016_si_001.pdf [file sc2c07016_si_001.pdf]

SUPPORTING INFORMATION for

Pig slurry management producing N mineral concentrates: a full-scale case study

Axel Herrera[1], Giuliana D'Imporzano[1], Elisa Clagnan[1], Ambrogio Pigoli[1], Elena Bonadei[2], Erik Meers[3], Fabrizio Adani[1*]

[1]Gruppo Ricicla - DiSAA, Università degli Studi di Milano, Milano, Italy

[2]O.B. Di Orazio Brunelli e Figli – S.N.C., Via Adua 52, Orzinuovi, BS, Italy

[3]Department of Green Chemistry and Technology, Faculty of Bioscience Engineering, University of Ghent, Coupure Links 653, 9000 Ghent, Belgium

***Corresponding author:** fabrizio.adani@unimi.it

This Supporting Information contains:

N. 1 Figure (Figure S1)

N. pages: 3.

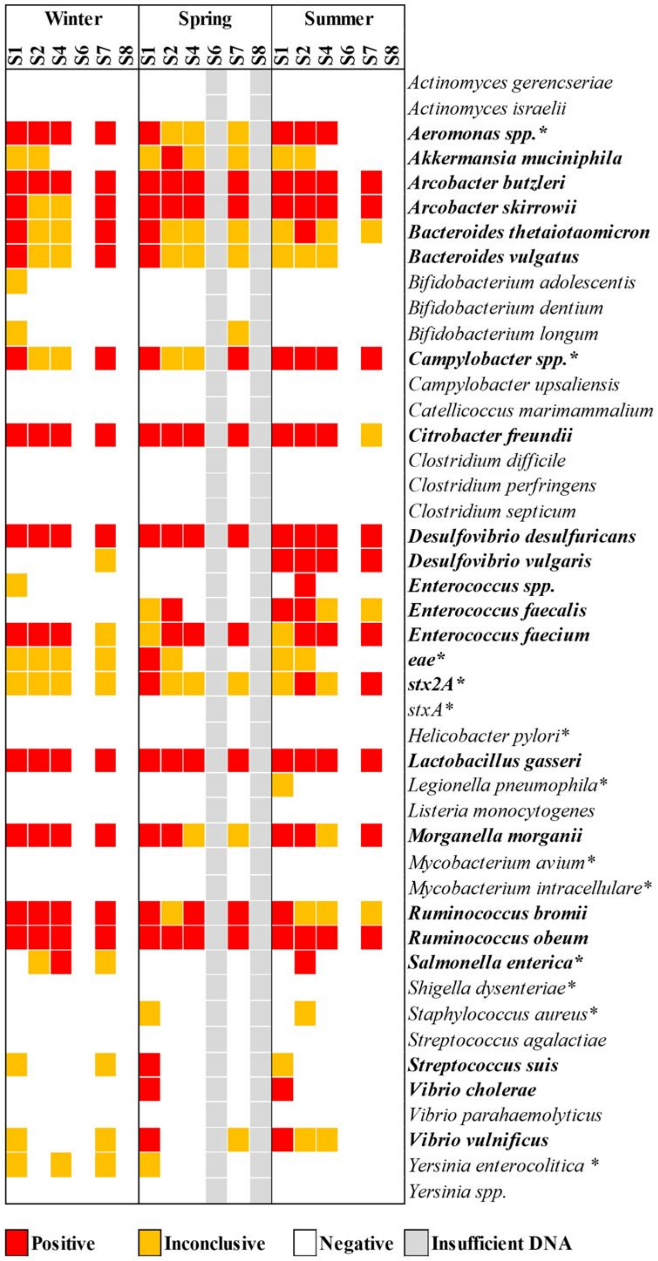

Figure S1. Enteric and pathogenic bacterial species and virulence markers screening in the solid-liquid fractions of interest: S1 (Slurry); S2 and S4 (Solid fractions); S3 (Liquid fraction after screw press); S6 (Liquid fraction after 1st RO stage); S7 (Concentrate) and S8 (Final permeate). Positive indicators in at least one sample are indicated in bold. *Highly important and emerging pathogens

in drinking water. *Aeromonas* spp. includes the species *A. enteropelogenes, A. hydrophila, A. punctata, A. media*; *Campylobacter* spp. includes *C. coli, C. subantarcticus, C. lari, C. jejuni*; *Enterococcus* spp. includes *E. gallinarum, E. casseliflavus*; *Yersinia* spp. includes *Y. pestis, Y. pseudotuberculosis*.
